# Supplementary material for: Associations between the rs5498 (A > G) and rs281432 (C > G) polymorphisms of the ICAM1 gene and atherosclerotic cardiovascular disease risk, including hypercholesterolemia
Source: PeerJ. 2022 Mar 8;10:e12972. doi: 10.7717/peerj.12972 (PMC8916030; doi:10.7717/peerj.12972)
Supplement: Table S1 — Abbreviation: ASCVD, Atherosclerotic cardiovascular disease. P value calculated using the χ2 test. ORa Unadjusted. ORb Adjusted for age, gender, BMI, smoking status, alcohol consumption status, and exercise habits. Pa value associated with odds ratio (OR)a. Pb value associated with ORb. P value <0.05 was considered statistically significant. [file peerj-10-12972-s001.docx]

**Supplementary Table 1**

**The effect of the interaction of the *ICAM1* rs281432 polymorphism and ASCVD risk factors**

| Variables | ASCVD risk factors | | *P* value | OR^a^ (95% CI) | *P^a^* value | OR^b^ (95% CI) | *P^b^* value |
| --- | --- | --- | --- | --- | --- | --- | --- |
|  | Low-risk | High-risk |  |  |  |  |  |
| 75^th^ percentile RHR (beats/min), n (%) | < 86 (n = 203) | ≥ 86 (n = 75) |  |  |  |  |  |
| *ICAM1* rs281432 (C > G) |  |  |  |  |  |  |  |
| CC | 82 (40.4) | 32 (42.7) |  | (Reference) |  | (Reference) |  |
| CG+GG | 121 (59.6) | 43 (57.3) | 0.732 | 0.91 (0.53-1.56) | 0.732 | 0.89 (0.56-1.55) | 0.690 |
| Tachycardia at RHR (beats/min), n (%) | ≤ 100 (n = 263) | > 100 (n = 15) |  |  |  |  |  |
| *ICAM1* rs281432 (C > G) |  |  |  |  |  |  |  |
| CC | 109 (41.4) | 5 (33.3) |  | (Reference) |  | (Reference) |  |
| CG+GG | 154 (58.6) | 10 (66.7) | 0.534 | 1.42 (0.47-4.26) | 0.536 | 1.46 (0.48-4.51) | 0.508 |
| BP (mmHg), n (%) | ≤ 130 and/or 85  (n = 221) | > 130 and/or 85  (n = 57) |  |  |  |  |  |
| *ICAM1* rs281432 (A > G) |  |  |  |  |  |  |  |
| CC | 88 (39.8) | 26 (45.6) |  | (Reference) |  | (Reference) |  |
| CG+GG | 133 (60.2) | 31 (54.4) | 0.428 | 0.79 (0.44-1.42) | 0.428 | 0.80 (0.44-1.48) | 0.484 |
| TC (mmol/L), n (%) | < 5.17 (n = 183) | ≥ 5.17 (n = 95) |  |  |  |  |  |
| *ICAM1* rs281432 (C > G) |  |  |  |  |  |  |  |
| CC | 70 (38.3) | 44 (46.3) |  | (Reference) |  | (Reference) |  |
| CG+GG | 113 (61.7) | 51 (53.7) | 0.195 | 0.72 (0.44-1.19) | 0.195 | 0.73 (0.44-1.22) | 0.225 |
| LDL-C (mmol/L), n (%) | < 3.36 (n = 205) | ≥ 3.36 (n = 73) |  |  |  |  |  |
| *ICAM1* rs281432 (C > G) |  |  |  |  |  |  |  |
| CC | 82 (40.0) | 32 (43.8) |  | (Reference) |  | (Reference) |  |
| CG+GG | 123 (60.0) | 41 (56.2) | 0.567 | 0.85 (0.50-1.47) | 0.567 | 0.87 (0.50-1.50) | 0.615 |
| HDL-C (mmol/L), n (%) | > 1.29 (women)  > 1.04 (men)  (n = 149) | ≤ 1.29 (women)  ≤ 1.04 (men)  (n = 129) |  |  |  |  |  |
| *ICAM1* rs281432 (C > G) |  |  |  |  |  |  |  |
| CC | 62 (41.6) | 52 (40.3) |  | (Reference) |  | (Reference) |  |
| CG+GG | 87 (58.4) | 77 (59.7) | 0.826 | 1.06 (0.65-1.70) | 0.826 | 1.08 (0.66-1.78) | 0.762 |
| TG (mmol/L), n (%) | < 1.69 (n = 192) | ≥ 1.69 (n = 86) |  |  |  |  |  |
| *ICAM1* rs281432 (C > G) |  |  |  |  |  |  |  |
| CC | 79 (41.1) | 35 (40.7) |  | (Reference) |  | (Reference) |  |
| CG+GG | 113 (58.9) | 51 (59.3) | 0.944 | 1.02 (0.61-1.71) | 0.944 | 1.12 (0.65-1.92) | 0.679 |
| TG/HDL-C (mmol/L), n (%) | < 1.75 (n = 211) | ≥ 1.75 (n = 67) |  |  |  |  |  |
| *ICAM1* rs281432 (C > G) |  |  |  |  |  |  |  |
| CC | 87 (41.2) | 27 (40.3) |  | (Reference) |  | (Reference) |  |
| CG+GG | 124 (58.8) | 40 (59.7) | 0.892 | 1.04 (0.59-1.82) | 0.892 | 1.10 (0.61-1.96) | 0.757 |

Abbreviation: ASCVD, Atherosclerotic cardiovascular disease.

*P* value calculated using the χ^2^ test. OR^a^ Unadjusted. OR^b^ Adjusted for age, gender, BMI, smoking status, alcohol consumption status, and exercise habits. *P^a^* value associated with odds ratio (OR)^a^. *P^b^* value associated with OR^b^. *P* value < 0.05 was considered statistically significant.
